# Factors Affecting Survival Outcome After Percutaneous Nephrostomy as Palliative Urinary Diversion in Obstructive Uropathy due to Advance Cervical Cancer Patients

**DOI:** 10.31557/APJCP.2021.22.4.1211

**Published:** 2021-04

**Authors:** Bambang Sasongko Noegroho, Andri Pratama Kurniawan, Zola Wijayanti, Akhmad Mustafa

**Affiliations:** *Department of Urology, Faculty of Medicine Universitas Padjadjaran/Hasan Sadikin General Hospital, Bandung, Indonesia. *

**Keywords:** Cervical cancer, obstructive uropathy, nephrostomy, survival rate

## Abstract

**Introduction:**

Cervical cancer is the 3^rd^ most common cancer in women. In late stages, obstructive uropathy due to mass infiltration is common and the mainstay of treatment for this condition is palliative urinary diversion through percutaneous nephrostomy. Nevertheless, complications due to nephrostomy may have adverse effects on some patients. Further study is necessary to determine whether nephrostomy is suitable for all cervical cancer patients with obstructive uropathy. This study aims to identify the determinants of survival rate of cervical cancer patients undergoing nephrostomy for obstructive uropathy and determine the group of cervical cancer patients that would benefit the most from nephrostomy.

**Methods:**

Data were obtained from medical records of cervical cancer patients in Hasan Sadikin Central Public Hospital from January 2018 to December 2019. Log-rank analysis was performed to assess the survival rate of patients based on clinical conditions (age, metastasis, and ECOG performance status) and initial laboratory results (hemoglobin, leukocyte, thrombocyte and blood acidity).

**Results:**

A total of 163 cases were identified from the medical records, with a median survival of 5(1-17) months. The results of the analysis showed that the survival rates of cervical cancer patients undergoing nephrostomy were significantly affected by age (p = 0.0001), metastasis (p = 0.0001), and ECOG performance status (p = 0.0001), while laboratory findings were not significant factors affecting survival (pHb=0.501; pLeu=0.634; pTr=0.077; pBGA=0.687).

**Conclusion:**

The survival after nephrostomy in advanced cervical cancer patients is largely affected by age, metastasis, and performance status. The choices of doing nephrostomy in those patients should be considering those factors to maximize the benefit over the risk of complications.

## Introduction

Cervical cancer is among the most common malignancies in females, ranking from the third most common in 2008 to the fourth most common malignancy in women in 2018. (Sherris et al., 2001; Arbyn et al., 2011). Globally, the annual incidence of cervical cancer is 13.1 per 100.000 females, with 311,000 deaths attributed to cervical cancer in 2018. About 80% of deaths caused by cervical cancer occurred in developing countries (Sherris et al., 2001). In Indonesia, cervical cancer is the second most common malignancy in females, with approximately 32,400 new cases diagnosed in 2018. (International Agency for Research on Cancer, 2019). Low-grade (localized) cervical cancer has a good prognosis, with a 5-year survival rate of 84.5% to 85.9% (Benard et al., 2017). Nevertheless, a large number of cervical cancer cases present at higher grades, due to inadequate screening, cultural practices, health-seeking behaviors, and limitations of the healthcare system (Mlange et al., 2015; Dunyo et al., 2018). A study (Dunyo et al., 2018) showed that nearly one-third of cervical cancer patients presented at advanced stages. Metastatic cervical cancer carries a poor prognosis, with an overall 5-year survival rate of 16.5% (Ferlay et al., 2013).

In addition, the five-year survival rate of stages I, II, III, IV cervical cancer in Indonesia were approximately 50%, 40%, 20%, and 0% respectively (Azis, 2009) 

Advanced cervical cancer patients often present with several life-threatening complications, such as kidney failure, thrombosis, and severe hemorrhages (Janaki et al., 2010; Tsai et al., 2012; Eleje et al., 2015). Urinary tract obstruction due to cervical cancer mass accounts for about 11-44% of complications in cervical cancer patients (Perri, 2019). Obstructive uropathy in cervical cancer may progress into hydronephrosis and kidney failure, both of which carry a poor prognosis. In these patients, severe complications of obstructive uropathy, including sepsis and uremia, may cause further deterioration of the patient’s condition and even death (Pergialiotis et al., 2019). 

The mainstay of treatment for obstructive uropathy caused by malignancy is urinary diversion through percutaneous nephrostomy or stenting. These procedures are considered safe and effective in alleviating symptoms associated with obstructive uropathy (Mishra et al., 2009). Although urinary diversion procedures can certainly improve the quality of life of advanced cervical cancer patients, current evidence suggests that urinary diversion does not significantly increase the survival rate of cervical cancer patients (Pergialiotis et al., 2019). In addition, several complications, although rare, have been associated with the nephrostomy procedure in cervical cancer, including pneumothorax, bowel injury, pararenal abscess, pyelonephritis, and urinoma (Van Aardt et al., 2017). This study, therefore, aims to identify the determinants of survival rate in cervical cancer patients undergoing nephrostomy for obstructive uropathy, and identify the subset of patients that would benefit the most from this procedure.

## Materials and Methods

This study is an observational, analytic, retrospective cross-sectional study. Data were obtained from medical records of cervical cancer patients undergoing nephrostomy for obstructive uropathy in Hasan Sadikin Central Public Hospital, Bandung. Data collection was limited to patients admitted from January 1, 2018 to December 31, 2019. Only medical records with a complete follow-up until patient death were included in the analysis.

*Statistical Analysis*

Descriptive data obtained include age, metastasis status, ECOG performance status, hemoglobin level, leukocyte count, thrombocyte count, blood pH, and eGFR stages. 

Survival rate analysis was performed using a log-rank test for nonparametric data, with patient’s age on diagnosis, staging, and ECOG functional status as independent variables and survival length as a dependent variable. This test compares the entire survival experience between groups with different independent factors, and then checks whether the survival curves are identical or not. Survival curves are estimated for each group, considered separately, using the Kaplan-Meier method. The confidence range used in this study was 95%, so the statistical analysis was considered significant if p-value < 0.05. Data analysis was performed using Statistical Package for Social Sciences (SPSS) version 23.0.

**Table 1:** Patient Demographics

Age	N (%)	Mean
<40	25 (15,33 %)	
40-60	117 (71,77 %)	49±9.38
>60	21 (12,88 %)	
Metastasis status		
Locally advanced	148 (90,79%)	
Distant metastasis	15 (9,2%)	
ECOG performance status		
0	0 (0.00%)	
1	0 (0.00%)	
2	16 (9,81 %)	
3	54 (33,12%)	
4	93 (57,05 %)	
Hemoglobin Level		
<5 gr/dl	18 (11%)	8.533±2.295 gr/dl
5-10 gr/dl	118 (72%)	
>10 gr/dl	27 (16.5%)	
Leukocyte count		
3,500/mm^3^	0 (0%)	16,718±9,029/mm^3^
3,500-1,2000/mm^3^	57 (35%)	
12,000-20,000/mm^3^	61 (37.4%)	
>20,000/mm^3^	45 (27.6%)	
Thrombocyte		
<150,000	14 (8.5%)	357,326±117,256/mm^3^
150,000-450,000	102 (62.5%)	
>450,000	47 (28.8%)	
Blood Gas Analysis (pH)		
Normal	112 (68.7%)	
Metabolic Acidosis (<7.35)	51 (31.3%)	
eGFR		
Stage 1	0 (0%)	
Stage 2	41 (25.1%)	
Stage 3	44 (26.7%)	
Stage 4	37 (22.7%)	
Stage 5	41 (25.1%)	

**Table 2:** Survival Rate of the Patients

Survival	N	%	Median
1 month	145	88.95%	5 (1-17) mo.
3 months	105	64.40%	
6 months	56	34.40%	
12 months	7	4.30%	
18 months	0	0.00%	

Kaplan-meier

**Figure 1:**
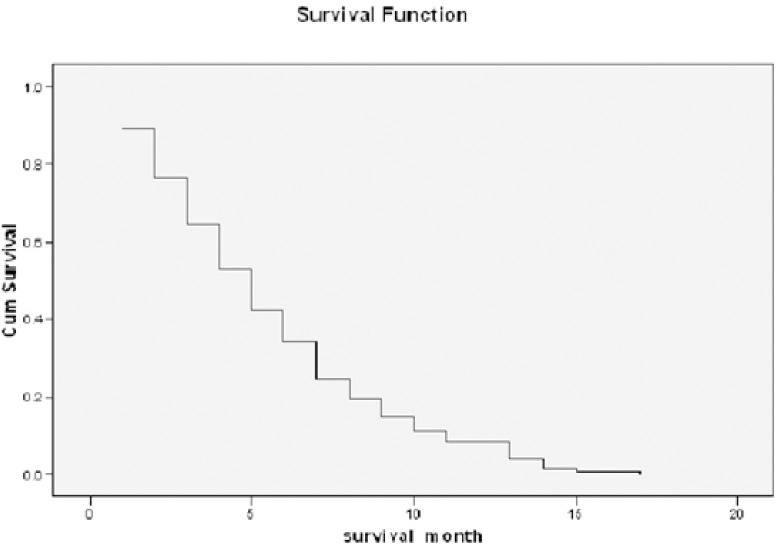
Kaplan-Meier Curve of the Patients

**Figure 2:**
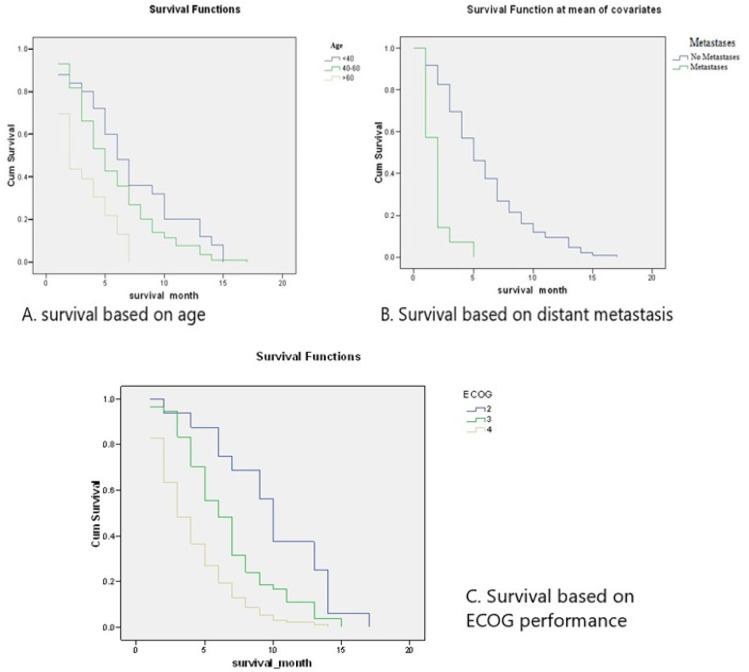
Kaplan-Meier Curve of Study Patients based on Clinical Findings

**Table 3:** Survival Rates of Study Patients Based on Clinical Findings

Age	N	Median	P*
<40 years	25	6 (1-15) mo.	0.0001
40-60 years	115	5 (1-17) mo.	
>60 years	23	2 (1-7) mo.	
Metastasis status			
Metastasis	14	2 (1-5) mo.	0.0001
No Metastasis	149	5 (1-17) mo.	
ECOG			
2	16	10 (2-17) mo.	0.0001
3	54	6 (1-15) mo.	
4	93	3 (1-14) mo.	

*Log-rank analysis

**Figure 3:**
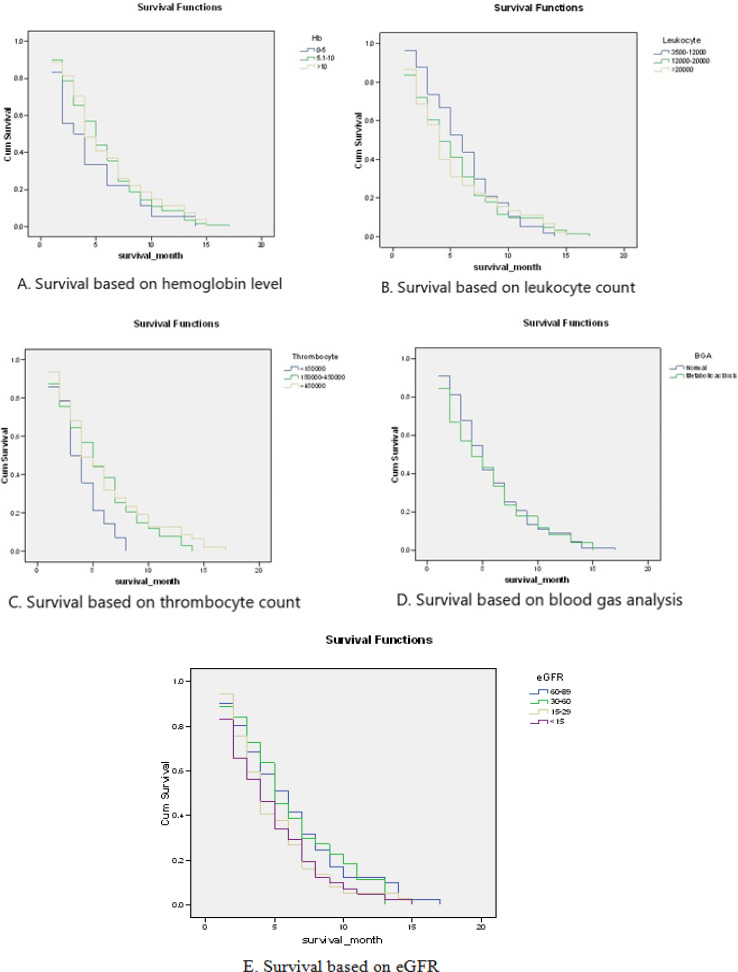
Kaplan-Meier Curve of Study Patients based on Laboratory Results

**Table 4:** Survival Rates of Patients based on Laboratory Results

	N	Median survival	P*
Hb			
<5 gr/dl	18	3 (1-14) months	0.501
5.1-10 gr/dl	118	5 (1-17) months	
>10 gr/dl	27	4 (1-15) months	
Leukocyte count			
3,500/mm^3^	0	-	0.634
3,500-12,000/mm^3^	57	6 (1-14) months	
12,000-20,000/mm^3^	61	4 (1-17) months	
>20,000/mm^3^	45	4 (1-15) months	
Thrombocyte count			
<150000	14	3 (1-8) months	0.077
150000-450000	102	5 (1-14) months	
>450000	47	4 (1-17) months	
Blood Gas Analysis (pH)			
Normal (7.35-7.45)	112	5 (1-17) months	0.687
Metabolic Acidosis (<7.35)	51	4 (1-15) months	
eGFR			
Stage 1	0		
Stage 2	41	6 (1-17) months	0.224
Stage 3	44	5 (1-13) months	
Stage 4	37	4 (1-15) months	
Stage 5	41	4 (1-15) months	

*Log-rank analysis

## Results

Total of 163 patients were obtained during the study period (Table 1). Based on age, 25 patients (15.33%) was aged below 40 years old, 117 patients (71.77%) was aged 40-60 years old, and 21 patients aged over 60 years old (12.88%). Most patients presented with locally advanced cervical cancer (148 patients, 90.79%). All patients had an ECOG score of at least 2, with the majority of patients having ECOG score 4 or severely disabled (93 patients, 57.05%).

Among these cases, the longest survival was 17 months. 6-month and 1-year survival rates were 34.4% and 4.3%, respectively. Survival rates obtained from the data are described in Table 2. A Kaplan-Meier survival function was generated from this data and is shown in Figure 1.

Based on age, the majority of patients in this study were aged 40-60 years old during diagnosis (115 patients, 70.55%), with a median survival rate of 6 months for patients under 40 years old, 5 months for patients aged 40-60 years old, and 2 months for patients older than 60 years old. A log-rank comparison of the groups using the Mantel-Cox test showed a chi-square value of 19.642 with a p-value of 0.0001 (p < 0.01). 

Fourteen patients (8.59%) in this study have metastatic disease. The median survival length for the metastatic patients was 2 (1-5) months, and the median survival length for non-metastatic patients was 5 (1-17) months. A comparison between the two groups showed a p-value of 0.0001 (p < 0.01), representing a highly significant difference between the two groups. The results of the survival analysis based on metastasis are shown in Table 4 and Figure 3.

Based on ECOG performance status, the majority of patients in this study were severely disabled, with 93 patients (57.06%) having an ECOG status of 4. The median survival rate for patients with ECOG status 2 was 10 (2-17) months, ECOG status 3 was 6 (1-15) months, and ECOG status 4 was 3 (1-14 months). A log-rank comparison of the groups using the Mantel-Cox test showed a chi-square value of 35.815 with a p-value of 0.0001 (p < 0.01). The results of the survival analysis based on ECOG performance status are shown in Table 5 and Figure 4.

## Discussion

Obstructive uropathy is one of the more common complications of advanced cervical cancer. Uropathy may be caused by external compression by the cervical mass, or malignant extension of cervical cancer to the urinary tract. This obstruction may cause uremia, electrolyte imbalance, hydronephrosis, and renal failure, which contributes significantly to the morbidity and mortality caused by cervical cancer (Mishra, 2009; Pergialiotis et al., 2019). In patients with obstructive uropathy with functional kidneys, aggressive treatment using urinary diversion procedures are required to prevent progressive kidney damage and subsequent uremia. In addition, uropathy relief through percutaneous nephrostomy or double-J stenting may be considered prior to chemotherapy to enable otherwise unfit patients to undergo chemotherapy after their kidney function improves (Lee et al., 1994; Mishra et al., 2009; Janaki et al., 2010).

Currently, there is limited data on the optimal urinary diversion method selection for cervical cancer patients. Several studies have shown that urinary diversion can improve renal function in cervical cancer patients, although the effects of the procedure on overall survival rates are unclear (Dientsmann et al., 2008; Pergialiotis et al., 2019; Maguire et al., 2020). Lapitan and Buckley (2011) showed that palliative urinary diversion may be beneficial for short-term chance of survival in cervical cancer patients. Patient age on cervical cancer diagnosis is significantly associated with higher rates of survival and higher success rates for percutaneous nephrostomy (Showalter et al., 2016). In a study (Van Aardt et al., 2017) younger patients have higher PCN insertion rates and better response to treatment, possibly due to a better metabolic reserve. In a small study of 15 patients (Mahajan et al., 2017) advanced age is a poor prognostic factor in advanced cervical cancer patients undergoing PCN, and the relief of obstructive uropathy in older patients is not associated with improved outcomes, even if technically feasible. 

Metastatic cervical cancer and performance status on presentation are known to significantly affect the survival rate of cervical cancer patients. Survival rates are much lower in metastatic (stage IV) cervical cancer compared to lower stages. 5-year survival rates range from 5.1% to 25% for stage IV cervical cancer, compared to as high as 100% for stages IA and IB cervical cancer ( Jayant et al., 2016; Hu et al., 2017; Espenel et al., 2019). Obstructive uropathy is common in advanced cervical cancer, although kidney metastasis is comparatively rare (Fan et al., 2015). In addition, obstructive uropathy and hydronephrosis may be caused by ureteral metastasis of cervical cancer (Kawanishi et al., 2019). A landmark cohort study by Perri et al., (2019) showed that several other factors also affect the survival rate of cervical cancer patients after nephrostomy, including ECOG performance status of over 1, diabetes mellitus, and ascites. The study also indicated that urinary diversion may not be beneficial in such patients. Moreover, significant complications were reported in 14-53% of these patients undergoing urinary diversion, including pyelonephritis, sepsis, and severe hematuria (Perri et al., 2019). In our study, metastatic disease and low performance status is associated with significantly lower survival rates. 

In conclusion, older age, metastatic disease, and lower ECOG performance status adversely affect survival rates in cervical cancer patients undergoing nephrostomy. The risks and benefits of obstructive uropathy relief through urinary diversion should be weighed carefully in these patients. 

## Author Contribution Statement

None.
